# Exploration of the Product Specificity of chitosanase CsnMY002 and Mutants Using Molecular Dynamics Simulations

**DOI:** 10.3390/molecules28031048

**Published:** 2023-01-20

**Authors:** Jianzhang Lu, Chu Wang, Yingying Ma, Kaifeng Liu, Xueqi Fu, Shu Xing

**Affiliations:** 1 Edmond H. Fischer Signal Transduction Laboratory, School of Life Sciences, Jilin University, Changchun 130012, China; 2 University of Edinburgh Institute (ZJU-UoE Institute), Zhejiang University, Haining 314400, China; 3 Key Laboratory for Molecular Enzymology and Engineering of Ministry of Education, School of Life Sciences, Jilin University, Changchun 130012, China

**Keywords:** chitosanase CsnMY002, molecular docking, MM-PBSA, molecular dynamics simulations, conformational changes

## Abstract

Chitosanase CsnMY002 is a new type of enzyme isolated from *Bacillus subtilis* that is used to prepare chitosan oligosaccharide. Although mutants G21R and G21K could increase Chitosan yield and thus increase the commercial value of the final product, the mechanism by which this happens is not known. Herein, we used molecular dynamics simulations to explore the conformational changes in CsnMY002 wild type and mutants when they bind substrates. The binding of substrate changed the conformation of protein, stretching and deforming the active and catalytic region. Additionally, the mutants caused different binding modes and catalysis, resulting in different degrees of polymerization of the final Chitooligosaccharide degradation product. Finally, Arg37, Ile145 ~ Gly148 and Trp204 are important catalytic residues of CsnMY002. Our study provides a basis for the engineering of chitosanases.

## 1. Introduction

Chitosan oligosaccharide (Cos) is a product of Chitosan degradation [1,2] with several biological properties [3,4], such as anti-oxidation [5,6], anti-cancer [7] or reducing blood fat [8].

At present, the best method of Cos preparation is enzymatic hydrolysis of Chitosan, which is mediated by chitosanases [9,10,11]. These are a group of enzymes with high similarity formed by seven families: GH3, GH5, GH7, GH8, GH46, GH75 and GH80 [12,13], where the GH46 family is different from the others.

Chitosanase MY002 (a GH46 family member) was successfully isolated in 2021 from *Bacillus subtilis* [14] and was referred to as chitosanase CSNMY002. Three mutants were produced, E19A, G21K and G21R, that were also active, but different from the wild type with respect to substrate binding and cleavage mechanism. The cleavage mode of Chitohexose (GlcN)_6_ by CSNMY002 is a “3 + 3” symmetry mode, whereas the three mutants have a different mechanism.

Subsequent functional experiments explored the enzymatic properties of these enzymes, but how these three mutations affect the substrate binding and splitting mechanism is not known. Herein, we have used molecular dynamics to simulate the reaction of CSNMY002 wild type (WT) and its two mutants G21R and G21K with Chitodisaccharide (GlcN)_2_ and Chitohexose (GlcN)_6_. Our results provide the basis for the design of new chitosanases.

## 2. Results and Discussion

### 2.1. The Binding Mode of (GlcN)_2_ to CSNMY002

**Figure 1:**
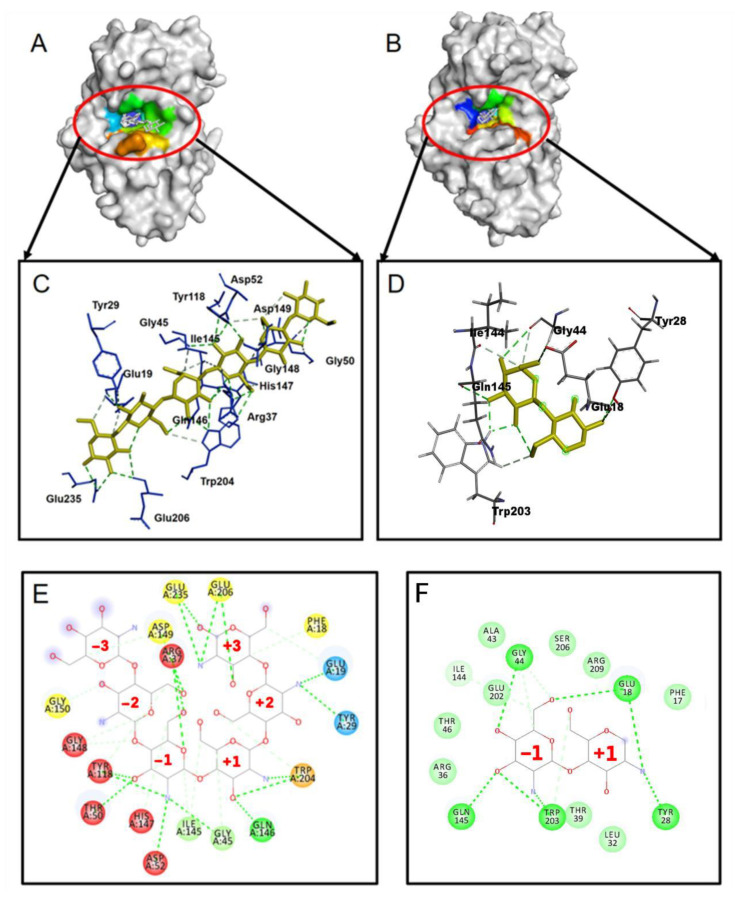
(**A**) Docking pose of (GLCN)_6_; (**B**) docking pose of (GlcN)_2_; (**C**) residues involved in (GLCN)_6_ binding; (**D**) residues involved in (GLCN)_2_ binding; (**E**,**F**) subsites for (GlcN)_6_; (**E**) and (GlcN)_2_ (**F**) binding: subsite −1 (red), subsite +1 (black).

The docking of (GlcN)_2_ and (GlcN)_6_ to WT CSNMY002 (Figure 1A–F) shows that both ligands have the same binding pose, with residues Gly45, IIe145, Gln146 and Trp204 involved in subsite +1 and Arg37, Gly45, Thr50, Asp52, Tyr118, IIe145, His147 and Gly148 involved in subsite −1.

### 2.2. System Stabilization

**Figure 2:**
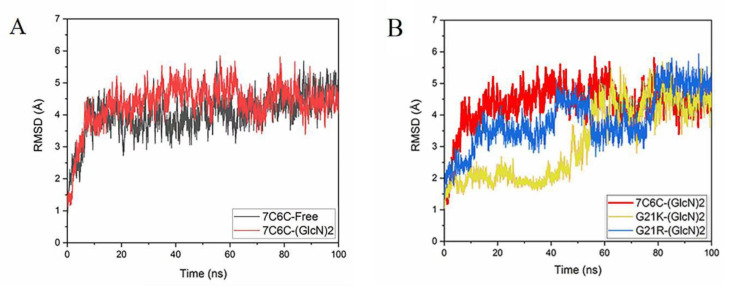
(**A**) RMSD plot of 7C6C-free and 7C6C-(GlcN)_2_ complex; (**B**) RMSD plot of 7C6C-(GlcN)_2_, G21K-(GlcN)_2_ and G21R-(GlcN)_2_.

The root-mean-square deviation (RMSD) values of the atomic skeletons in the four simulation architectures (Figure 2A,B) showed that the four reaction systems reached equilibrium after 60 ns. The final RMSD was always below 5 Å, indicating that the systems were stable during the 100 ns MD simulation.

**Figure 3:**
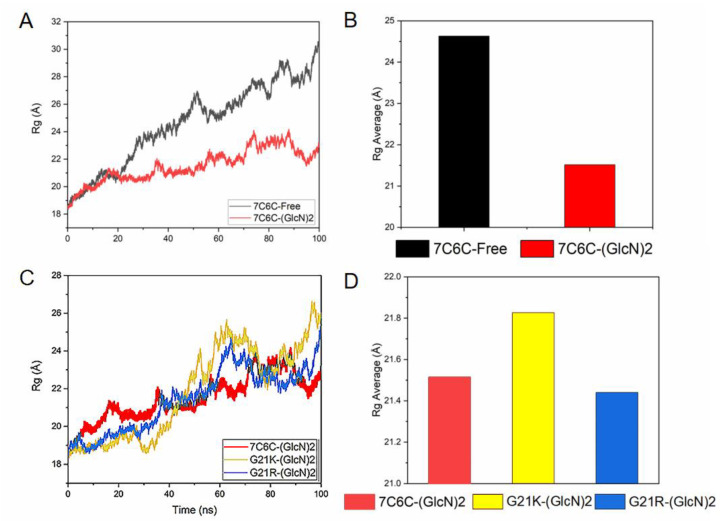
(**A**) Rg plot of 7C6C-free and 7C6C-(GlcN)_2_ complex; (**B**) average of Rg plots of 7C6C-free and 7C6C-(GlcN)_2_ complexes during MD simulations; (**C**) Rg plot of 7C6C-(GlcN)_2_, G21K-(GlcN) and G21R-(GlcN)_2_; (**D**) average of Rg plots of 7C6C-(GlcN)_2_, G21K-(GlcN) and G21R-(GlcN)_2_.

Then Rg value represents the tight degree of protein structure. The smaller the value of Rg is, the more closely the 3D structure of the protein is. The Rg was below 30 Å in all four systems (Figure 3A–D), and it was smaller in the mutants, indicating a conformational change after binding to ligand.

**Figure 4:**
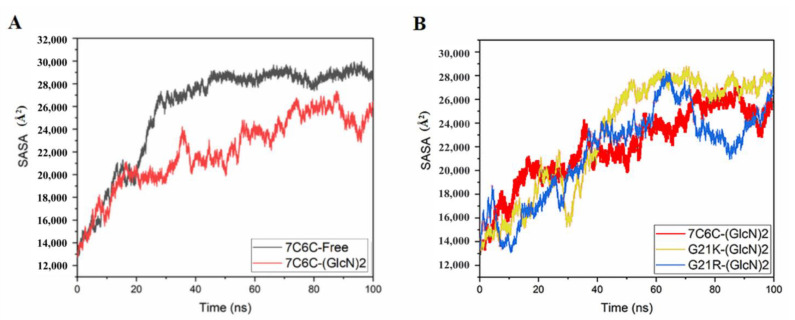
SASA plots. (**A**) 7C6C-free (black) and 7C6C-(GlcN)_2_ (red); (**B**) 7C6C-(GlcN)_2_ (red), G21K-(GlcN)_2_ (yellow) and G21R-(GlcN)_2_ (blue).

**Figure 5:**
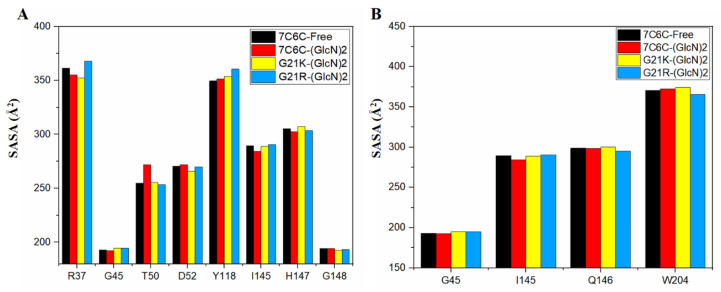
(**A**,**B**) SASA plot of each residue in subsite −1 (**A**) and subsite +1 (**B**).

The SASA value of the free protein was stable (around 28,000~30,000 Å^2^) after 100 ns (Figure 4 and Figure 5), but it was smaller in the three complexes, with final values stable between 26,000 and 28,000 Å^2^, suggesting reduced protein hydrophilicity caused by ligand binding. The SASA residue values of subsites −1 and +1 (Figure 5A,B) showed a significant increase in Thr50 in 7C6C-(GlcN)_2_, compared to 7C6C-free, but residues at subsite 1 did not show significant differences. The SASA values of residue Tyr118 in the two mutants increased, whereas G21K did not show significant changes in these key sites.

Overall, the four systems were stable after 100 ns MD simulations and could be used in subsequent steps.

### 2.3. Conformational Changes between Protein and Ligands

**Figure 6:**
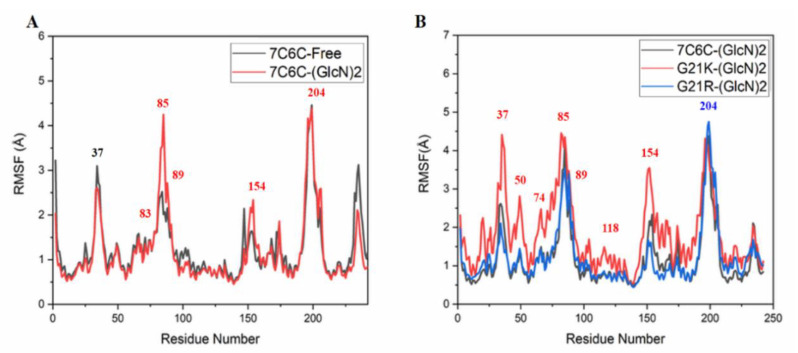
RMSF plots. (**A**) 7C6C-Free (black) and 7c6c-(GLCN)_2_ (red); (**B**) 7c6c-(GLCN)_2_ (black), G21k-(GLCN)_2_ (red) and G21R-(GLCN)_2_ (blue).

Compared with free CSNMY002, binding to (GLCN)_2_ induced changes in residues 83–89 (Figure 6A), and differences were observed in the active site region. The RMSF value of the G21R mutant and free WT were almost the same and only showed a strong fluctuation at the active site Trp204. In contrast, the G21K mutant showed strong fluctuations during the MD simulations at Arg37, Thr50, Tyr118 and Trp204.

**Figure 7:**
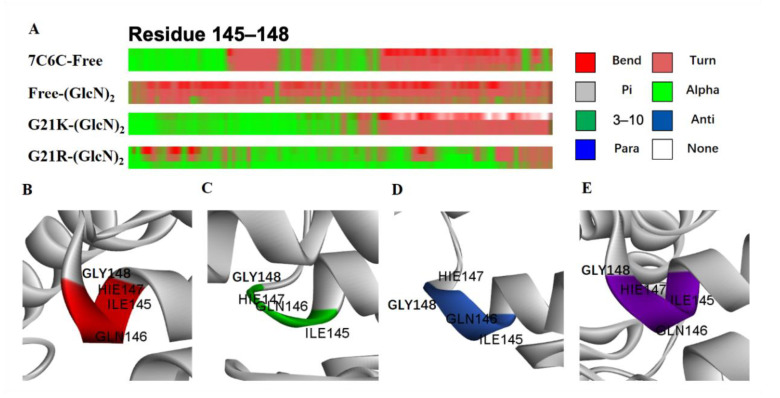
(**A**) Secondary structure changes in residues Ile145-Gly148; (**B**) 7C6C-free; (**C**) 7C6C-(GlcN)_2_; (**D**) G21K-(GlcN)_2_; (**E**) G21R-(GlcN)_2_.

The secondary structure change probability is shown in (Figure 7A–E).

In two mutants, the α-helices remained unchanged in the Ile145-Gly148 domain (Table 1). In the free WT, the α-helices in the inner part dropped and sharply formed a loop. Residues Ile145-Gly148 located at the α6 region contain key catalytic residues at subsites −1 and +1. In both mutants, the enhanced helix probability may increase the tunnel length of the α6 helix, which may facilitate sliding of the substrate into the tunnel.

**Figure 8:**
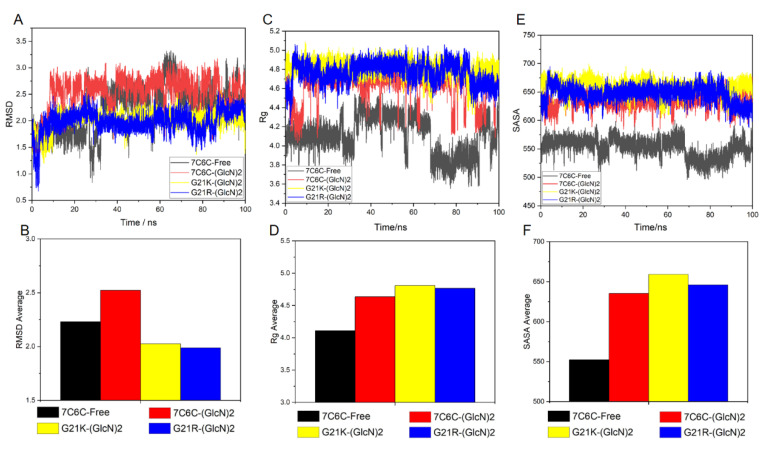
Analysis of active residues in Ile145-Gly148 fragment of each reaction system. (**A**) RMSD; (**B**) RMSD average; (**C**) Rg; (**D**) Rg average; (**E**) SASA; (**F**) SASA average.

The amino acid residues of the Ile145-Gly148 fragment were used to analyze RMSD, Rg and SASA. The RMSD value of the free protein fluctuated, especially around 60 ns (Figure 8A,B), but that of the three complexes was more stable. The Rg of the free protein in this region was smaller than in the three complexes (Figure 8C,D), where it increased after binding the substrate, especially those involving the two mutant complex systems. The SASA of the free protein in this region was lower, whereas the hydrophilicity of the three complex systems increased. The SASA values of the two mutants showed an increase (Figure 8E,F).

**Figure 9:**
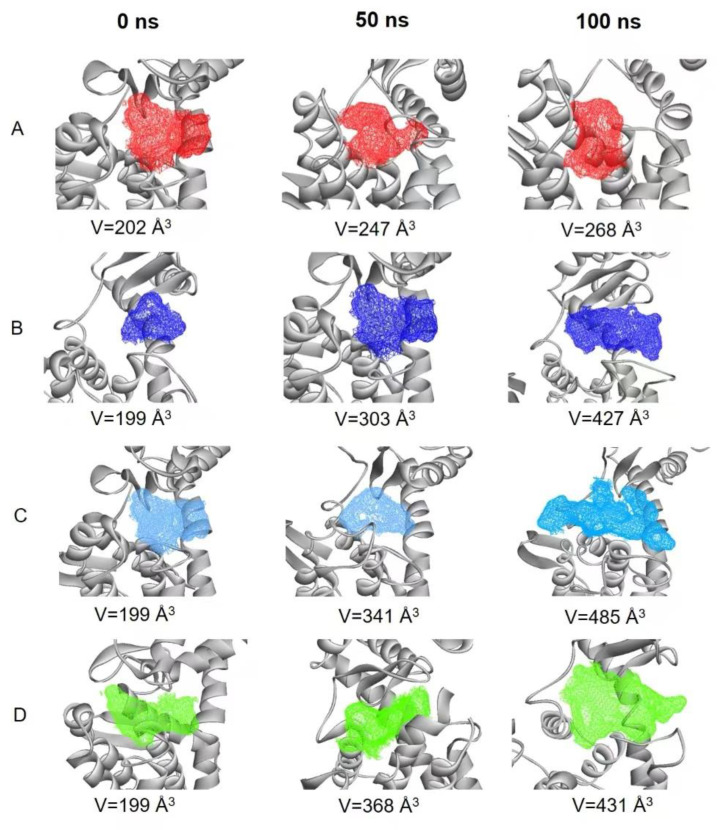
Size and shape of active pockets at 0, 50 and 100 ns in: (**A**) free 7C6C (red); (**B**) 7C6C-(GlcN)_2_ (dark blue); (**C**) G21K-(GlcN)_2_ (light blue); (**D**) G21R-(GlcN)_2_ (green).

Protein active pocket analysis is important to study protease activity. The active pocket region in the MD simulation was calculated using POCASA [15]. Changes in active pocket volume were examined after 0, 50 and 100 ns (Figure 9A–D). Compared with the free protein, the active pocket volumes of the three complex systems increased. In the two mutants, they were bigger than in the 7C6C-(GlcN)_2_ complex, which may be useful for the substrate to slide into and to start the catalytic reaction. This may be one of the reasons for the improvement of enzyme activity in the two mutants.

### 2.4. Cross-Correlation Analysis

**Figure 10:**
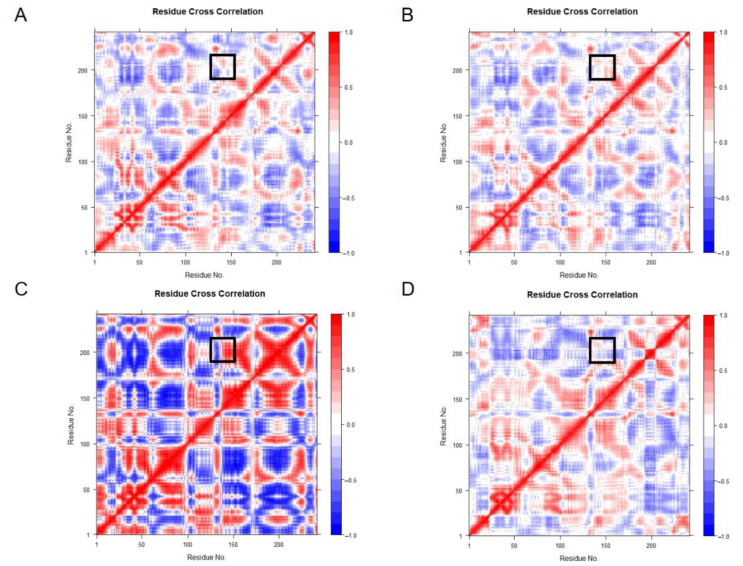
Correlation diagram of residue covariance used to mark the interaction positions of segments Ile145-Gly148 and His198-Val208 with black rectangles: (**A**) 7C6C-Free; (**B**) 7C6C-(GlcN)_2_; (**C**) G21K-(GlcN)_2_; (**D**) G21R-(GlcN)_2_.

Cross-correlation matrix analysis can find protein regions that experience large conformational changes. The G21K-(GlcN)_2_ complex showed more flexibility in the MD simulations, whereas Ile145-Gly148 were negatively correlated in the G21K-(GlcN)_2_ system (Figure 10A–D).

### 2.5. MM-PBSA Calculation

To confirm these results, the binding free energies of the protein–ligand complexes in 7C6C-(GlcN)_2_, G21K-(GlcN)_2_ and G21R-(GlcN)_2_ were calculated with MM-PBSA, showing nonbonded van der Waals (ΔE_vdW_), nonbonded electrostatic (ΔE_ele_) interactions and binding free energy (ΔG_bind_) (Table 2). Mutants showed lower binding free energy, indicating more favorable binding to substrates and improved catalytic ability, confirming previous experimental results.

## 3. Methods

### 3.1. System Preparation

The structure of chitosanase CSNMY002 (PDB: 7C6C) [14] was obtained from the protein database www.rcsb.org (accessed on 21 December 2021). The structures of Chitodisaccharide (GlcN)_2_ and Chitohexose (GlcN)_6_ were downloaded from the PubChem database (https://PubChem.ncbi.nlm.nih.gov (accessed on 21st December 2021)) (PUBCHEM CID: 91859334, 100978292) [16]. After removing water and ligand from the protein, mutations at residue 21 were introduced using Chimera Software [17].

### 3.2. Molecular Docking

The substrate was docked to CSNMY002 with AutoDock 4.2 software [18,19,20,21]. The grid size was set to 60 × 50 × 60 Å and spacing between grid points was 0.375 Å. After docking, protein–ligand complexes with the lowest energy were selected and used for subsequent molecular dynamics simulations.

### 3.3. Molecular Dynamics Simulations

Amber 16 software [22,23] was used to simulate the systems consisting of free WT, WT-(GlcN)_2_, G21K-(GlcN)_2_ and G21R-(GLCN)_2_ for 100 ns. The force field for the protein was Amber FF99SB [24,25], whereas for (GLCN)_2_ it was GAFF2 [26,27]. The TIP3P model [28,29] was used, and periodic boundary conditions were applied to the reaction system during the simulation. Because the net charge in the initial reaction system is not zero, Na^+^ was added in the initial stage of the simulation. The information of each system is listed in Table 3.

After the system was built, it was energy-minimized using the steepest descent and a conjugate gradient method, each with 500 steps. After this minimization, the initial structure was stable. The temperature of the simulated reaction was raised from 0 to 300 K in 50 ps. At the end of heating, the system was left to react for another 50 ps. Finally, the system was equilibrated with constant pressure under NPT condition [30,31,32], with a constant pressure balance of 500 ps at 300 K. This was the last step for the system balance, which took 2 fs. After stabilization of all the thermodynamic parameters, a 100 ns MD simulation was performed for each system, collecting data every 1 fs, with a storage interval of 2 ps/interval and a total of 10,000 frames.

AutoDockTools 1.5.6 was used for the six molecular docking systems. The results of molecular docking were visualized with Pymol 2.4.0. Data from four MD systems were collected and analyzed for protein structure fluctuation, combined with analysis of pocket volume, stretch kinetics and secondary structure. Trajectory analyses were computed using Amber16′s CPPTRAJ module [15] and included RMSD, radius of gyration, RMSF, SASA and dictionary of secondary structures. The cross-correlation matrix of the trajectory was generated with Tcl script in VMD, and its eigenvector and eigenvalue were calculated [33,34].

### 3.4. MM-PBSA

The MM-PBSA method [35] was used to predict binding free energies and relative stabilities of the models [36]. Binding free energies were calculated using the MM-PBSA method in AMBER 16. A total of 100 snapshots were chosen evenly from the MD trajectory. Total binding energy (ΔG_bind_) was computed using the equation:

ΔG_bind_ = G_complex_ − (G_protein_ + G_ligand_) (1)

where ΔGbind is the binding free energy between protein and ligand, calculated as the difference between the total free energy of the complex (Gcomplex) and the sum of the free energy of protein (Gprotein) and ligand (Gligand). The binding energy is expressed as the combination of enthalpy and entropy terms:

ΔG_bind_ = ΔH − TΔS (2)

where TΔS refers to the entropic contribution to the free energy in a vacuum, and T and S are temperature and entropy, respectively. The changes in protein and ligand upon binding were similar in all complexes, with very small entropy differences; therefore, the calculation of the solvate entropy term is omitted.

ΔH = ΔE_MM_ + ΔG_solvation_ (3)

where E_MM_ is the molecular mechanics energy of the molecule expressed as the sum of internal energy and electrostatic and van der Waals energies.

ΔE_MM_ = ΔE_vdw_ + ΔE_ele_ (4)

The solvation free energy is the sum of polar and nonpolar contributions:

G_solvation_ = G_polar_ + G_nonpolar_ (5)

where G_nonpolar_ is calculated from the solvent-accessible surface area (SASA):

G_nonpolar_ = γ SASA + b (6)

Here, γ = 0.0072 kcal/mol/Å, and b = 0 kcal/mol.

## 4. Conclusions

First, binding of substrate can activate the protein, changing the conformation of the active and catalytic regions. The combination of chitosaccharide and chitosanase can effectively stabilize the structure of chitosanase during the reaction and enhance its stability. The point mutation of residue 21 changed the original properties of regions 145–148 and 198–208. When combined with the substrate, the segment underwent obvious stretching deformation, thus changing the initial wave condition. In chitosanase CSNMY002, the active region recruits Chitosan molecules through stretching and deformation. When Chitodisaccharide or Chitohexose docks and reacts with CSNMY002, a tight interaction network forms, and Chitosan is degraded.

Second, point mutations such as G21K and G21R lead to conformational changes of the original degradation site and a changed degree of polymerization of the final degradation product. In the two mutants, the helical augmentation effect effectively inhibited the catalytic action of the original specificity on the +1/−1 site and at the same time increased the tunnel length of the α6 helix, which made the hydrogen bond network between chitosanase and chitosan molecules more stable and closer.

Finally, in this enzyme, Arg37, Ile145-Gly148 and Trp204 are important catalytic residues, and Arg37 forms two stable hydrogen bonds with the -1 site which helps to form a tighter complex with Chitosan. Ile145-Gly148 is an important binding and catalytic site, which can catalytically degrade the β-(1,4)-glycosidic bond under the synergistic action of Glu19 sites while binding the +1/−1 sites. Trp204, located in the α9 helix, is also an important site for degradation, which can be induced by the synergistic action of Lys21 or Arg21. These sites are potential target sites for CSNMY002 optimization.

## Figures and Tables

**Table 1:** Probability of α-helix in Ile145-Gly148 during 100 ns MD simulations.

	7C6C-Free		7C6C-(GlcN)_2_		G21K-(GlcN)_2_		G21R-(GlcN)_2_	
Residue	α-helix	Loop	α-helix	Loop	α-helix	Loop	α-helix	Loop
**Ile145**	0.42	0.58	0.24	0.76	0.49	0.51	0.62	0.38
**Gln146**	0.38	0.62	0.08	0.92	0.49	0.51	0.59	0.41
**His147**	0.37	0.63	0.08	0.92	0.45	0.35	0.64	0.36
**Gly148**	0	0.01	0	0.01	0	0.01	0	0.01

**Table 2:** Results of MM-PBSA.

	7C6C-(GlcN)_2_	G21R-(GlcN)_2_	G21K-(GlcN)_2_
ΔE_vdW_	−24.04 ± 1.04	−24.53 ± 0.73	−23.03 ± 1.11
ΔE_ele_	0.43 ± 1.13	−3.37 ± 0.74	−1.34 ± 1.04
ΔE_PB_	12.18 ± 0.58	15.72 ± 1.19	9.90 ± 1.53
ΔG_gas_	−23.62 ± 0.90	−27.90 ± 1.20	−24.37 ± 1.78
ΔG_solv_	12.18 ± 0.58	15.72 ± 1.89	9.90 ± 1.53
ΔG_bind_	−11.44 ± 1.04	−12.19 ± 0.86	−14.47 ± 0.47

**Table 3:** Details corresponding to the four systems.

Complex	Protein	Ligand	Ions	Total Molecules
**7C6C-Free**	1	None	Na^+^ (22)	13,158
**7C6C-(GlcN)_2_**	1	(GlcN)_2_	Na^+^ (23)	13,155
**G21K-(GlcN)_2_**	1	(GlcN)_2_	Na^+^ (24)	13,149
**G21R-(GlcN)_2_**	1	(GlcN)_2_	Na^+^ (18)	13,148

## Data Availability

Not applicable.
